# Glabellar vertical line as a reference goal for anteroposterior maxillary position

**DOI:** 10.1590/2177-6709.24.3.45.e1-5.onl

**Published:** 2019

**Authors:** Marcos J. Carruitero, Ximena M. Ambrosio-Vallejos, Carlos Flores-Mir

**Affiliations:** 1 Antenor Orrego Private University (Trujillo, Peru).; 2 Private practice (Trujillo, Peru).; 3 University of Alberta, Division of Orthodontics (Edmonton, Canada).

**Keywords:** Facial profile, Diagnosis, Orthodontics, Orthognathic surgery

## Abstract

**Objective:**

The aim of this study was to evaluate the use of glabellar vertical line (GVL) as the anteroposterior maxillary position goal.

**Methods:**

A cross-sectional study was conducted assessing 129 participants (20.21 ± 1.99 years): 67 women (20.16 ± 1.99 years), and 62 males (20.26 ± 2.06 years). The facial profile photographs were taken with a posed smile in natural head position. The linear distance from the most facial convexity of the upper central incisor (FA) to the goal anterior-limit line (GALL) and also from FA to GVL were measured and compared. Wilcoxon signed-ranks test was applied. To determine the correlation between the distances, Spearman’s correlation coefficient was used. Simple and multiple linear regression were also performed.

**Results:**

The GALL-GVL separation was 0.54 ± 1.14 mm (95%CI: 0.34-0.74). A strong correlation between FA-GALL and FA-GVL distances (Spearman’s rho=0.983 [95%CI: 0.976-0.988], *p*< 0.01) was identified. The FA-GVL distance explains almost all the total variation of FA-GALL (R^2^=95.84%, *p*< 0.01). The FA-GALL distance can be predicted by using the formula: FA-GALL=0.5+0.9*(FA-GVL).

**Conclusion:**

These findings suggest that GVL could be used as an easier-to-use treatment goal to determine the maxillary anteroposterior position, compared to GALL, to improve facial harmony profile goals in cases where the maxillary incisors are properly positioned anteroposteriorly.

## RESUMO

**Objetivo:**

o objetivo desse estudo foi avaliar o uso da linha vertical glabelar (GVL) como referência para a posição anteroposterior da maxila.

**Métodos:**

esse estudo transversal avaliou 129 pacientes (20,21 ± 1,99 anos), sendo 67 mulheres (20,16 ± 1,99 anos) e 62 homens (20,26 ± 2,06 anos). As fotografias de perfil foram realizadas com os pacientes em sorriso posado e em posição natural de cabeça. As distâncias lineares entre a porção mais convexa da face vestibular do incisivo central superior (FA) e a linha do limite anterior (GALL), e entre a FA e a GVL foram mensuradas e comparadas. Para a análise comparativa, utilizou-se o teste de postos sinalizados de Wilcoxon. O coeficiente de correlação de Spearman avaliou a correlação. Além disso, foi realizada regressão linear múltipla.

**Resultados:**

a distância entre GALL e GVL foi de 0,54 ± 1,14mm (95% IC: 0,34-0,74). Uma forte correlação foi identificada entre as distâncias FA-GALL e FA-GVL (Spearman’s rho = 0,983 (95%IC: 0,976-0,988), *p*< 0,01). A distância FA-GVL contempla quase todas as variações de FA-GALL (R^2^ = 95,84%, *p*< 0,01). Essa distância pode ser calculada através da fórmula: FA-GALL= 0,5 + 0,9*(FA-GVL).

**Conclusão:**

os resultados encontrados sugerem que a GVL pode ser adotada como uma referência de fácil utilização na determinação da posição anteroposterior da maxila, quando comparada à GALL para a melhora do perfil facial nos casos em que os incisivos superiores estão corretamente posicionados.

## INTRODUCTION

Orthodontic and orthognathic treatment planning focus on obtaining an optimum facial profile,^1^ ^-^ ^3^ and the maxillary anteroposterior position has a clear effect on facial profile esthetics.^4^ Independently of the methods and indicators already proposed for measuring maxillary anteroposterior position,^5^ ^-^ ^8^ publications on facial profile attractiveness and the impact of maxillary anteroposterior position are still in continuous development.^9^ ^-^ ^11^

Within facial analysis, the glabella point has been considered as a reference since the beginning of our specialty. In 1900, Edward H. Angle^12^ discussed his “line of harmony”, a vertical line that touches Glabella, Subnasale, and Pogonion in the profile “with perfect harmony.” Later, in the 1980s, Andrews^13^ ^-^ ^15^ undertook a research project to search for data-based treatment goals for maxillary anteroposterior position using Glabella and other points assessed in posed smile.

Andrews^15^ used the forehead as a basis for evaluating the maxillary anteroposterior position using the Element II concept. According to this concept, the ideal position of the maxilla is when the FA point - clinical midpoint of the vestibular surface of upper incisor - contacts the goal anterior-limit line (GALL), a parallel line to the frontal plane of the head passing through a line between the forehead’s anterior-limit line (FALL) and the glabellar vertical line (GVL). FALL and GVL are parallel to the frontal plane of the head, FALL pass through the forehead’s facial-axis (FFA) point and GVL pass through glabella. The FFA point is clinically determined according to the type of forehead: for straight forehead it is located between Trichion and Glabella, for angular and rounded foreheads it is located between Superion - a point near to Trichion at the prominent upper region of the forehead - and Glabella.^14^ ^-^ ^16^

The correct maxillary anteroposterior position occurs when the FA point of the upper central incisor is located between FALL and GVL, ideally coinciding with GALL, which becomes a treatment goal. When FA is forward GVL, there is maxillary protrusion.^15^ FA is located close to GALL and GVL, which means that the distances FA-GALL and FA-GVL use to be similar. So, GVL may also be considered as a goal anterior-limit for the maxillary AP position, which would be important because Glabella is easier to locate than GALL and is also located in a minimally variable area of the face, which has been considered a useful landmark for assessing the facial profile.^16^

Of the two distances, FA-GALL and FA-GVL, the first one corresponds to the gold standard. Dr. Andrews considered GALL as the ideal aesthetic anteroposterior location of the maxilla.^16^ Nevertheless, to locate GALL it is necessary to make previous steps: determine the type of patient’s forehead; locate Trichion, which is not so simple to identify in all patients;^17^ locate Superion, which depends on the type of forehead; use a formula to locate GALL; and finally, if GALL comes out of GVL, this line should be considered as GALL.^16^ Based on this, it may be more convenient to simply use GVL from the beginning.

Thereby, the purpose of this study was to evaluate the mean differences and the correlation between FA-GALL and FA-GVL distances. The alternative hypothesis was that the FA-GALL and FA-GVL distances are similar and correlated.

## MATERIAL AND METHODS

Ethics and protocol was approved by the Stomatology Permanent Research Committee of the Antenor Orrego Private University (Trujillo, Peru). The ethical approval number was 12602014-FMEHU-UPAO.

### Study sample

This study counted with a sample of 129 participants (20.21 ± 1.99 years), 67 women (20.16 ± 1.99 years) and 62 males (20.26 ± 2.06 years). The sample was constituted by volunteer students of a local university from Trujillo-Peru, who met the selection criteria. All participants signed an informed consent. The sample size was calculated using the correlation found between scores of the FA-GALL and FA-GVL distances reported from a pilot study. A statistical power of 80% and a confidence level of 95% were considered. 

Inclusion criteria were: male or female participant, natural dentition, no missing anterior teeth, any type of malocclusion but with the upper maxillary central incisors clinically considered in an ideal position, with favorable buccolingual inclination related to the occlusal plane.^18^ Exclusion criteria were: background of forehead or maxillofacial trauma, orthodontic, orthopedic or orthognathic treatment.

### Determination of GALL

Trichion (T), Superion (S), Glabella (G) and the FFA point of the forehead were identified on the midsagittal plane of the head, as described by Andrews.^16^ Marks with small balls of modeling clay of about 1 mm in diameter were placed on the forehead at T, S, G, and FFA points, in order to visualize them in the photography. Once the points were located, the linear measure over the forehead from T to G (T-G) and from S to G (S-G) were recorded. These linear measures were used for standardizing the photographs before printing them.

**Figure 1:**
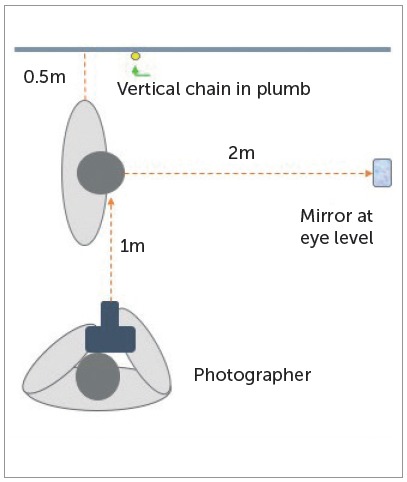
Schematic representation for facial profile photographs in natural head position.

**Figure 2:**
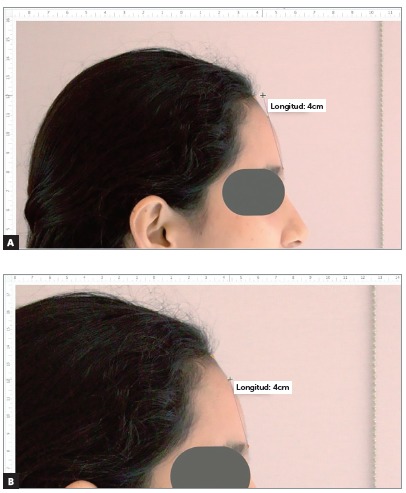
Standardization of profile photographs using Power Point; A) photograph during standardization setting the real Superion-Glabella (S-G) dimension; B) after enlarging the image until reach the real measurement of the distance S-G.

The facial profile photographs were taken with a posed smile in natural head position, standardized as a position of the head in an upright head posture with the eyes focusing a point in the distance, at eye level.^19^ ^,^ ^20^ A vertical chain in plumb line was placed near to the subject, being the true vertical line (TVL) (Fig 1). Photographs were standardized using the Power Point program (Microsoft PowerPoint version 2010) using the Format and Height of Form options, enlarging or reducing the image until reach the measurement of the distances T-G or S-G (Fig 2). 

**Figure 3:**
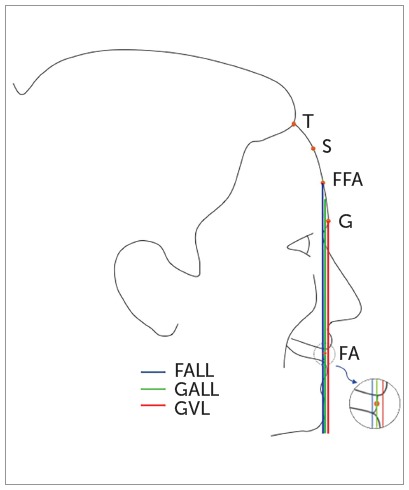
Points and linear measurements: Trichion (T), Superion (S), Glabella (G), the Forehead’s Facial-Axis (FFA) point, the Facial Axis (FA) point of the upper central incisor, the Goal Anterior-Limit Line (GALL), and the Glabellar Vertical Line (GVL).

On each printed standardized photograph, the forehead inclination (FI) was traced to determine GALL, as proposed by Andrews.^16^ This line and GVL were traced parallel to the TVL (Fig 3).

### Measurement of FA-GALL and FA-GVL distances

On each printed photograph, the horizontal linear separation in millimeters from the FA point to the GALL line was measured and it was considered as the FA-GALL distance (control group). Similarly, the horizontal linear separation in millimeters from the FA point to the GVL line was also measured, and considered as the FA-GVL distance (study group).

### Method error

To evaluate the method error, measurements in 10 photographs not considered in the final study were assessed. The forehead identification and the FA-GALL and FA-GVL measurements were carried out by the same researcher twice (with a two weeks interval) in order to evaluate intraexaminer reliability. To assess the interexaminer reliability, the same cases were evaluated by another researcher (Table 1).

**Table 1:** Intra and interexaminer reliability of the variables (n=10).

Variable	Calibration	Statistical test	95% confidence interval	P value
		Kappa		
Type of forehead	Intraexaminer	0.836	(0.444-1.000)	<0.001*
	Interexaminer	1.000	(1.000-1.000)	<0.001*
		ICC		
FA-GVL distance	intraexaminer	0.929	(0.729-0.982)	<0.001*
	interexaminer	0.959	(0.837-0.990)	<0.001*
FFA -FA distance	intraexaminer	0.962	(0.853-0.990)	<0.001*
	interexaminer	0.976	(0.905-0.994)	<0.001*
Forehead inclination	intraexaminer	0.998	(0.991-1.000)	<0.001*
	interexaminer	0.996	(0.985-0.999)	<0.001*
FA-GVL distance	intraexaminer	0.928	(0.596-0.983)	<0.001*
	interexaminer	0.960	(0.844-0.990)	<0.001*
FA-GALL distance	intraexaminer	0.998	(0.991-1.000)	<0.001*
	interexaminer	0.996	(0.985-0.999)	<0.001*

FFA = forehead’s facial-axis; FA = facial axis of the maxillary central incisor; GVL = glabellar vertical line; FALL = forehead anterior-limit line; ICC = Intraclass correlation coefficient; Kappa = weighted kappa coefficient. *Significant at p < 0.01.

### Statistical analysis

Data were processed using the statistical software Stata v. 13 (StataCorp LP, Texas, USA). Means, standard deviations, and the 95% confidence interval values were calculated. The agreement between the observations of the type of forehead was performed by using the weighted kappa coefficient. Tracings were evaluated by the intraclass correlation coefficient test. Before making any comparisons, compliance with the assumptions of normality and homogeneity of variances with Shapiro-Wilk and Levene’s test were evaluated. The FA-GALL group had normal distribution, but the FA-GVL group and the GALL-GVL were not normally distributed; therefore, Wilcoxon signed-ranks test was applied. To determine the correlation between FA-GALL and FA-GVL distances, Spearman’s correlation coefficient was used. A simple linear regression analysis was used to evaluate the possibility of predicting the FA-GALL distance using the FA-GVL distance. An analysis of multiple linear regression, in which age, sex, and FI were included as factors, was also performed. Statistical significance was set at 5% in all tests.

## RESULTS

Reliability was considered adequate. High concordance with values greater than 0.836 were found (Table 1).

The mean difference between FA-GALL and FA-GVL (GALL-GVL distance) in the total sample was 0.54±1.14 mm; in females was 0.64±1.25 mm; in males was 0.44±0.99 mm; in the 18- to 20-year-old group was 0.56±1.13 mm; and in the 22- to 24-year-old group was 0.51±1.16 mm. Comparing the distances between FA-GALL and FA-GVL, it was found statistically significant differences in all cases (*p*< 0.01) (Table 2).

**Table 2:** Mean differences and correlation between FA-GALL and FA-GVL distances.

Groups		Distance	n	Mean (mm)	SD	Mean difference (FA-GALL) - (FA-GVL)/GALL-GVL distance	SD of the mean difference	95% Confidence Interval of the difference	P value	Spearman correlation coefficient	95% Confidence Interval of the correlation	P value
Total		FA-GALL	129	0,09	4,78	0.54	1.14	(0.34-0.74)	<0.001^ⱡ^	0.983	(0.976 -0.988)	<0.001*
		FA-GVL	129	-0,44	5.26							
Sex	Female	FA-GALL	67	-0,28	5,08	0,64	1,25	(0.33-0.94)	<0.001^ⱡ^	0.981	(0.969-0.988)	<0.001*
		FA-GVL	67	-0,92	5,63							
	Male	FA-GALL	62	0,5	4,43	0,44	0,99	(0.18-0.70)	<0.001^ⱡ^	0.993	(0.972-0.990)	<0.001*
		FA-GVL	62	0,86	4,83							
Age groups	18 to 21 years	FA-GALL	87	-0,3	4,81	0,56	1,13	(0.31-0.80)	<0.001^ⱡ^	0.980	(0.969 -0.987)	<0.001*
		FA-GVL	87	-0,86	5,3							
	22 to 24 years	FA-GALL	42	0,91	4,66	0.51	1,16	(0.15-0.87)	0.002^ⱡ^	0.986	(0.975 -0.993)	<0.001*
	FA-GVL	42	0,4	5,16								

FA = facial axis of the central upper incisor; GALL = Goal anterior-limit line; GVL = glabellar vertical line; FA-GALL = distance from FA to GALL; FA-GVL = distance from FA to GVL; SD = standard deviation. ^ⱡ^Wilcoxon signed-ranks test, significant at P <0.01. *Significant at P <0.01.

A strong correlation between FA-GALL and FA-GVL distances was found (*p*< 0.01), with correlation coefficients of 0.983 (95% CI: 0.976-0.988) in the total sample, 0.981 (95% CI: 0.969-0.988) in females, 0.993 in males (95% CI: 0.972-0.990), 0.980 (95% CI: 0.969-0.987) in the 18- to 20-year-old group, and 0.986 (95% CI: 0.975-0.993) in the 22- to 24-year-old group (Table 2). 

By simple regression analysis, the following formula was developed to predict the FA-GALL distance using the FA-GVL distance; the R^2^ was 95.84%, *p*< 0.01:

FA−GALL=0.49+0.89*(FA−GVL)

By multiple regression analysis, it was observed that the GALL-GVL distance was not associated with sex, age, and the type of forehead (R^2^=1.38%, *p*= 0.628). 

## DISCUSSION

It has been reported that the use of the forehead is a useful method to evaluate the maxillary anteroposterior position,^16^ suggesting that its ideal position occurs when the upper central incisor profile is located at GALL, between FALL and GVL.^14^ ^,^ ^16^ However, from all forehead’s landmarks used, Glabella seems to be the easiest to identify, since the other points require additional location considerations that could hinder its identification, particularly the Trichion point.^17^ The procedure for its location requires to have the forehead free of hair or even the use of a hairband to retract the hair back, procedure that could be uncomfortable for patients; likewise, it is difficult to accurately identify the hairline, especially if patients have started to become bold.

With the purpose of avoiding these relative difficulties, a possible alternative is to consider Glabella as a reference goal. In this study, the mean difference between FA-GALL and FA-GVL was close to 0.5 mm. If that “mean difference” refers to the GALL-GVL distance and, by definition, GALL cannot be anterior to GVL,^15^ the present results would indicate that GALL must be always close to GVL. Andrews^16^ found that when the FA point was located forward to Glabella, the jaws showed no facial harmony; so, if FA is anterior to GVL, the maxilla would be in an advanced position. According to the present study, the relatively small distance between GALL and GVL could be a good reason for considering maxillary optimum anteroposterior position in a range of 0.5 to 1 mm behind GVL. 

On the other hand, no normal distribution was found for most of the summarized values, so this relatively wide dispersion of data could explain that there were large standard deviations for FA-GALL, FA-GVL, and GALL-GVL distances. So, further research considering facial profile aesthetics analysis based on GVL by a layperson jury is suggested.

The results of this study showed a strong correlation between FA-GALL and FA-GVL. The FA point tended to move backward or forward similarly from GALL or GVL. This behavior was repeated in subgroup analysis: females, males, and groups based on ages. The forehead acted as a neutral, fixed and invariable reference. Thus, since GVL does not depend on the type or inclination of the forehead, it will be an invariable reference too.

During a complementary analysis, a multiple linear regression analysis showed no influence of sex, age, and the type of forehead. So, it would not be necessary to have these features previously. If greater precision in the GALL location is desired, the formula found in the present study could be used [FA-GALL=0.5+0.9*(FA-GVL)], by means of which the FA-GALL distance could be predicted by knowing FA-GVL, since this latter distance would be easier to identify (considering negative value if FA-GVL is behind GVL). The coefficient of determination was relatively high as it will explain around 100% (95.84%) of the total variance.

Ideally, GALL is expected to be located between FFA and glabella (between FALL and GVL), which has been the starting point for various studies. Cao et al.^7^ assessed the effect of the maxillary incisor anteroposterior position on smiling profile esthetics moving them using a digital imaging program anteriorly and posteriorly to GALL by 1 to 4 mm, respectively. They concluded that harmonious smiling profiles with protrusion of maxillary incisors (FA ahead of GALL within 2 mm), would not negatively affect smiling profile aesthetics. This study suggests that it is possible to consider as aesthetically acceptable the location of FA ahead of GALL. In the present study, it was found that GALL tends to be located behind GVL, so if the FA point is nearby GVL, the aesthetics of the facial profile in smiling would not be negatively affected.

For Element II diagnosis, it is necessary to locate maxillary incisor anteroposterior position in millimeters relative the FALL line, to know how much movement is necessary to correct its location regarding the GALL line. This measurement is proposed to be performed clinically.^14^ In the present study, the measurement of FA-GALL and FA-GVL distances was not performed clinically. No instrument currently exists for clinical measurement of these distances. So, based on previous studies,^1^ ^,^ ^7^ ^-^ ^9^ ^,^ ^17^ it was decided to carry out the procedure in standardized photographs. It was also considered the standardized natural head position because any variation in the head position in the sagittal plane could affect the FA-GALL and FA-GVL distances, as well as their difference. The only limitation of this method was that involved 2-D, and not 3-D or animated profile images; nevertheless, the philosophy of the 6 elements contemplates the 2-D profile evaluation,^14^ so it was decided to follow this principle.

The findings of this study can be incorporated into routine orthodontic and orthognathic records, diagnosis, and treatment planning. Both the orthodontist and the orthognathic surgeon can perform the evaluation of the anteroposterior maxillary position analyzing the facial profile in smile, in order to observe the closeness of the upper incisors to the GVL, which could guide the clinician in the final decision of where to locate the maxilla at the end of the treatment. As Andrews^16^ indicates, it is important the addition of a smiling profile photograph, especially with the forehead showing glabella and with maxillary incisors fully exposed, so diagnostic records as well as clinical evaluation of the smiling facial profile will allow clinicians to document the orientation of the patients’ maxillary central incisors to the forehead. The findings suggest applying the GVL as a complementary treatment goal for young-adult patients seeking improved facial harmony.

## CONCLUSION

These findings suggest that GVL could be used as an easy-to-use treatment goal to determine the maxillary anteroposterior position, to improve facial harmony profile goals in cases where the maxillary incisors are properly positioned anteroposteriorly.
